# Examining the Relationship Between Trauma, Post‐Traumatic Stress Disorder and Psychosis in Patients in a UK Secondary Care Service

**DOI:** 10.1176/appi.prcp.20220028

**Published:** 2023-01-20

**Authors:** David Martin, Michelle Philips, Harriet Greenstone, Jonathan Davies, Guy Stewart, Elizabeth Ewins, Stan Zammit

**Affiliations:** ^1^ Centre for Academic Mental Health, Population Health Sciences Bristol Medical School University of Bristol Bristol UK; ^2^ Avon and Wiltshire Mental Health Partnership NHS Trust Bath UK; ^3^ MRC Centre for Neuropsychiatric Genetics and Genomics Division of Psychological Medicine and Clinical Neurosciences Cardiff University School of Medicine Cardiff UK

## Abstract

**Objective:**

Traumatic experiences and post‐traumatic stress disorder (PTSD) are common in schizophrenia. However, few studies screening for PTSD have established the temporality of PTSD‐related traumatic events to psychosis onset. Furthermore, it is unclear how many patients attribute a trauma‐based contribution to their psychosis or would find trauma‐focused therapy acceptable. We examine the prevalence and timing of trauma in psychosis, as well as patient views on the relationship between their trauma experiences and mental health difficulties, and on receiving trauma‐focused therapy.

**Methods:**

Sixty‐eight patients with an at‐risk mental state (ARMS) or psychotic disorder in a UK secondary‐care setting completed self‐report measures of trauma and PTSD, and undertook research interviews. Proportions and odds ratios were derived with 95% confidence intervals.

**Results:**

We recruited 68 participants (estimated response rate 62%; psychotic disorder *n* = 61, ARMS *n* = 7). Sixty three (95%) reported traumatic events and 32 (47%) reported childhood abuse. Twenty‐six individuals (38%) met criteria for PTSD, though for >95% this was not recorded in their notes, and 25 (37%) had sub‐threshold PTSD. For 69% of participants, their worst trauma occurred before the onset of their psychosis symptoms. Most (65%) believed their psychosis symptoms were related to past traumas and 82% of these were interested in receiving trauma‐focused therapy.

**Conclusions:**

PTSD is common in and often pre‐dates onset of psychosis. Most patients believe their symptoms and traumas are related and would be interested in trauma‐focused therapy if available. Studies evaluating the effectiveness of trauma‐focused therapies for those with or at high‐risk of psychosis are required.

## Highlights

- Post‐traumatic stress disorder (PTSD) is common in and often pre‐dates onset of psychosis.
- Most patients believe their symptoms and traumas are related and would be interested in trauma‐focused therapy if available.
- Studies evaluating the effectiveness of trauma‐focused therapies for those with or at high‐risk of psychosis are required.

Schizophrenia is one of the leading causes of disability worldwide (1) and imposes a high risk of suicide and increased mortality on sufferers (2). The efficacy of current treatments remains limited, and relapse rates remain high (3, 4, 5).

An increasing body of evidence supports a causal effect of trauma on psychotic experiences, psychotic disorders, and transition to psychosis (6, 7, 8, 9, 10). Individuals with psychotic illnesses also frequently have co‐morbid post‐traumatic stress disorder (PTSD). A systematic review found that 31% (95% CI 21%–41%) of patients with a psychotic disorder met criteria for PTSD on screening, and that this was almost all undetected by the clinical teams (i.e., no PTSD documentation in clinical records) (11). However, there was substantial heterogeneity in estimates, and >80% of studies had a moderate or high risk of bias, indicating that more rigorous studies are required to estimate how common PTSD is in people with psychosis.

Whilst co‐morbid PTSD is associated with poorer clinical outcomes in schizophrenia (12), there are also theoretical reasons to believe that psychotic experiences in some individuals might arise from mechanisms related to those underlying PTSD symptoms. Therefore, using evidence‐based trauma‐focused psychological interventions for PTSD (13) would theoretically reduce psychotic phenomena (14, 15). Hence high levels of unresolved PTSD symptoms therefore, if they exist, may represent a missed opportunity to reduce onset of psychosis in high‐risk individuals, and to improve outcomes and reduce relapse in those with psychotic illnesses.

Service user opinion on trauma‐focused treatment options is unclear but is important as endorsed by high‐level policy (16) and by evidence that belief in the type of treatment received affects outcomes (17).

Therefore, the aims of our study were to answer the following questions:

1. What is the prevalence of PTSD in secondary care service users with At Risk Mental State (ARMS) or psychosis?
2. What proportion of service users have experienced trauma prior to developing ARMS or psychosis?
3. What are services users' views on the relationship between their trauma experiences and their mental health difficulties?
4. Are individuals with ARMS and psychosis interested in receiving trauma‐focused therapies, and does this differ by the type of trauma experienced?

## METHOD

### Sample

This was a cross‐sectional study of participants recruited through the Avon and Wiltshire Partnership Mental Health NHS Trust (AWP) in the UK. Researchers screened clinical teams' caseload of patients within the Early Intervention (EI), Recovery, and Community Mental Health Teams, as well as inpatient wards to identify potentially eligible service users, and then liaised with service users' clinicians to ensure that they had capacity to give informed consent. Clinicians were asked to discuss the study with their patients and contact details of those who expressed an interest were then passed on to the research team.

Participants were eligible if they were aged 16 and over and had received a diagnosis of a psychotic disorder (ICD‐10 codes F20‐F29, F30.2, F31.2, F31.5, F32.3, F33.3), or met the operational criteria for ARMS according to the Comprehensive Assessment of At‐Risk Mental States (CAARMS) (18). Exclusion criteria included inadequate capacity to give informed consent; being at high risk of suicide or having attempted suicide in the previous 6 months; estimated IQ below 70; inadequate command of English language to complete questionnaires and participate in the interview.

Participants were invited to an appointment where capacity to consent was determined and written informed consent received. They were then asked to complete the study measures.

We estimated that a sample size of 70 would provide us with proportions and confidence intervals that even at their lower limits would include a clinically impactful result (e.g., *N* = 70, 30% undetected PTSD; proportion = 0.3, 95% CI 0.20–0.42).

The authors assert that all procedures contributing to this work comply with the ethical standards of the relevant national and institutional committees on human experimentation and with the Helsinki Declaration of 1975, as revised in 2008. All procedures involving human subjects/patients were approved by Southwest—Central Bristol Research Ethics Committee (Reference: 16/SW/0126), on June 7, 2016.

### Measures

#### Trauma

We used two self‐report questionnaires to measure trauma: the Life Events Checklist (LEC) for DSM‐5 (19) and the Childhood Trauma Questionnaire (CTQ) (20). Both questionnaires were adapted for the purposes of this study.

The LEC scale is comprised of 16 items which assess exposure to events such as accidents, and physical or sexual assault (items rated as: 0 = Doesn't apply, 1 = Not sure, 2 = Learned about it, 3 = Witnessed it, and 4 = Happened to me). In addition, we asked participants to report “any other very stressful event or experience” they experienced in the past.

The CTQ assesses physical, sexual, and emotional abuse, and physical and emotional neglect in childhood (items rated as: 1 = Never true, 2 = Rarely true, 3 = Sometimes true, 4 = Often true, 5 = Very often true). We used 10 of the original 28 questions, selecting two that were representative from each trauma type, to reduce participant burden (see Supplement S1). We derived a binary variable indexing exposure to any childhood abuse from the CTQ. As our primary measure, we rated as exposed those participants who scored at least four points on any of the 10 items. As a sensitivity analysis, we used a lower cut‐off, requiring exposed to score at least two points on any item.

#### PTSD

We used the PTSD Checklist for DSM‐5 (PCL‐5) (21) to assess PTSD symptoms. The 20 items assess four past‐month symptom clusters (re‐experiencing, avoidance, negative cognitions/mood, hyperarousal) (items rated as: 0 = Not at all, 1 = A little bit, 2 = Moderately, 3 = Quite a bit, 4 = Extremely). We derived a binary variable representing ‘Probable PTSD’ using a PCL‐5 cut‐off score of 33, (22), and also a provisional DSM‐V PTSD diagnosis (criteria A‐E) as used previously (2). We also derived a variable representing sub‐threshold PTSD, defined as meeting two or three of the DSM‐V B‐E criteria (23).

#### Additional questions

Those participants who reported traumatic experiences and had PTSD symptoms were asked additional questions about their views of the relationship between their trauma experience and their psychosis symptoms, and whether a trauma‐focused therapy would be of potential interest to them.

### Statistical Analysis

All analyses were carried out in Stata version 15. We examined i) the prevalence of trauma, ii) the prevalence of PTSD, iii) the proportion of people who believed that their mental health difficulties were related to past trauma, iv) the proportion of people who were interested in receiving a trauma‐focused therapy, and v) the association between trauma type and interest in receiving trauma‐focused therapy. We used logistic regression to examine the association between trauma type and willingness to receive trauma‐focused therapy. Odds ratios (OR), 95% confidence intervals (95% CI) and *p*‐values are reported.

## RESULTS

### Sample

**FIGURE 1:**
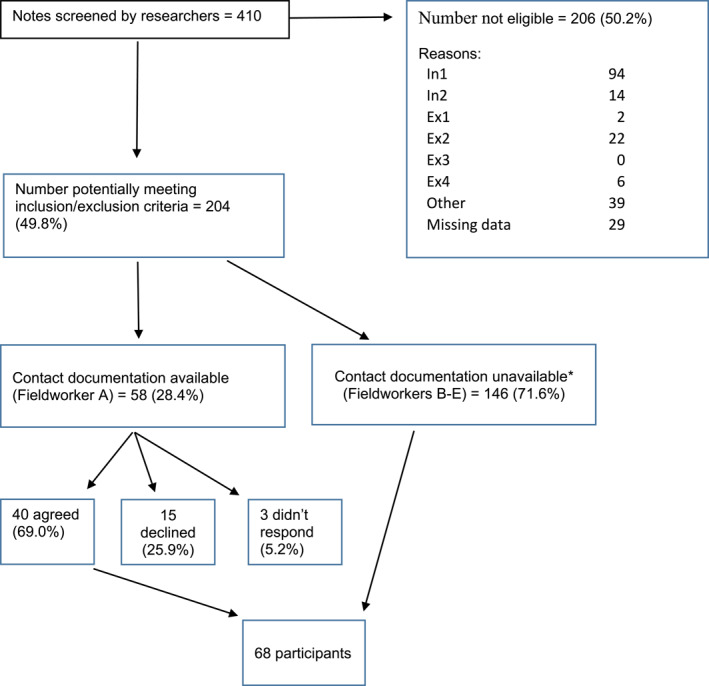
Recruitment flowchart. *Due to administrative error.

The clinical notes were screened of 410 people who were being supported by any team potentially working with adults with psychosis or ARMS. Of these, 204 (50%) were thought to be potentially eligible for inclusion, and 68 participants were recruited. Due to an administrative error we do not have a complete record of how many of those potentially eligible for inclusion were approached about the study. Based on the complete records available for one of the fieldworkers, 49 people responded out of 58 people contacted (84%), and 36 of these agreed to take part in the study (62% of those contacted). See Figure 1.

Of the study participants, 47 (69%) were recruited from the EI teams, eight (12%) from forensic inpatient wards, six (9%) from the community adult mental health teams, five (7%) from inpatient adult acute wards, and two (3%) from a mental health rehabilitation inpatient ward. Five (7%) were diagnosed with affective psychosis, 17 (25%) with schizophrenia, three (4%) with schizoaffective disorder, 29 (43%) with unspecified psychosis, five (7%) with brief psychosis, seven (10%) were identified as ARMS, and 2 (3%) were missing data on diagnosis.

### Trauma Prevalence

Based on the Life Events Checklist (LEC), 95% of study participants (*n* = 63, 95% CI 87%, 99%) reported a traumatic event that met DSM‐V criterion A for PTSD (Table 1). Of these, 98% (*n* = 62) were directly exposed to a trauma. Of those participants who experienced a trauma, 22% (*n* = 15) experienced one type of trauma, and 69% (*n* = 47) experienced at least two types of trauma.

**TABLE 1:** Number (%) of participants reporting traumatic events on the Life Events Checklist

	Happened to them	Witnessed it	Learnt about it	Part of job	Any	Happened to them or witnessed it
					*N* (%)	*N* (%)
Natural disaster	5	1	8	0	14 (21)	6 (9)
Fire/explosion	7	8	7	0	22 (32)	15 (22)
Transport accident	22	5	8	0	35 (51)	27 (40)
Serious accident	16	6	3	0	25 (37)	22 (32)
Toxic substance	8	0	2	0	10 (15)	8 (12)
Physical assault	43	9	4	1	57 (84)	52 (76)
Assault with weapon	21	8	5	1	35 (51)	29 (43)
Sexual assault	18	2	9	1	30 (44)	20 (29)
Other sexual	28	4	3	0	35 (51)	32 (47)
Combat or war‐zone	2	0	5	0	7 (10)	2 (3)
Captivity	12	1	6	0	19 (28)	13 (19)
Life‐threatening illness/injury	13	7	12	0	32 (47)	20 (29)
Severe human suffering	17	7	6	1	31 (46)	24 (35)
Sudden violent death	‐	8	9	0	17 (25)	8 (12)
Sudden accidental death	‐	8	7	0	15 (22)	8 (12)
Caused serious harm/death	8	2	1	0	11 (16)	10 (15)

Forty seven percentage (*n* = 32, 95% CI 35%, 59%) of study participants reported a history of childhood trauma on the CTQ (defined by rating at least one question as ‘often true’ or ‘very often true’). Thirty nine participants (57.4%) recorded one type of trauma, 16 (23.5%) recorded two types, nine (13.2%) recorded three types, one (1.5%) recorded four types, and three (4.4%) all five types. The most common form of childhood abuse reported was emotional abuse (29%), followed by sexual abuse (21%), physical abuse (18%), and physical and emotional neglect (approximately 15%) (Table 2).

**TABLE 2:** Prevalence (*n* (%)) of trauma type reported on CTQ

	Emotional abuse	Physical abuse	Sexual abuse	Emotional neglect	Physical neglect	Any trauma
Less stringent^a^	46 (68%)	26 (38%)	24 (35%)	36 (53%)	34 (50%)	55 (81%)
More stringent^b^	20 (29%)	12 (18%)	14 (21%)	10 (15%)	11 (16%)	32 (47%)

^a^ Participants who rated items as “Rarely true”, “Sometimes true”, “Often true” or “Always true” were classed as having had experienced a traumatic event.

^b^ Participants who rated items as “Often true” or “Always true” were classed as having had experienced a traumatic event.

When we applied less stringent criteria for coding childhood trauma, requiring the rating of at least one question as ‘rarely true’, the prevalence of childhood trauma increased to 80% (*n* = 55). The highest prevalence was for emotional abuse (29%), followed by emotional and physical neglect (approximately 50%), and physical and sexual abuse (approximately 35%). Sixteen participants (23.5%) recorded one type of trauma, 12 (17.7%) recorded two types, 15 (22.1%) recorded three types, 11 (16.2%) recorded four types, and 14 (20.6%) all five types.

### Timing of Most Traumatic Event and Psychosis Diagnosis

For 45 of 65 respondents (69.2%), the stated onset of their most stressful event was prior to the date of their first psychosis diagnosis on their clinical notes. For only four (6.2%) individuals was the stated onset after the date of diagnosis, and 16 (24.6%) participants had data missing on age at which their worst traumatic event occurred.

The mean age at which the participants' most stressful event occurred was 16.8 years (SD = 1.1) and the participant‐reported mean age at which they first sought help for their mental health difficulties was age 23.6 years (SD = 1.1).

The most common types of trauma reported as the participant's ‘worst traumatic event’ were: bereavement (*n* = 9, 13.2%), childhood abuse (*n* = 8, 13.2%) and their experience of psychosis (*n* = 8, 11.8%)

### Prevalence of PTSD Symptoms

**FIGURE 2:**
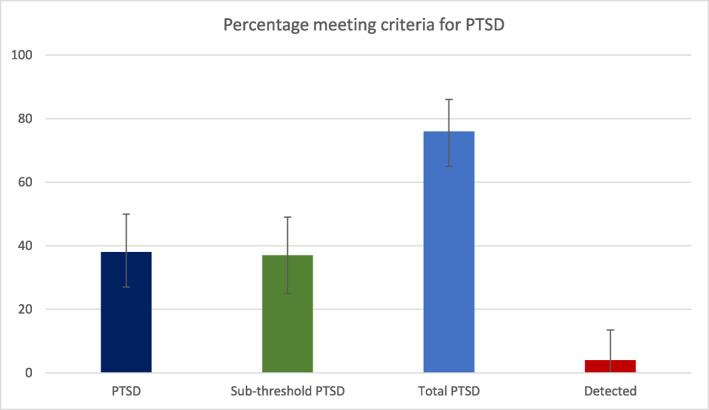
Percentage of participants (and 95% CI) who meet criteria for post‐traumatic stress disorder (PTSD), sub‐threshold PTSD, total PTSD, and detected by their clinical teams (i.e., a diagnosis was recorded in their notes).

Fifty‐one percent (*n* = 35, 95% CI 39%, 64%) of the sample met the criteria for ‘probable PTSD’, defined as a score of ≥33 on the PCL‐5. Applying DSM‐5 criteria to the PCL‐5, 38% (*n* = 26, 95% CI 27%, 51%) of participants met the criteria for a provisional diagnosis of PTSD, and an additional 37% (*n* = 25) met the criteria for sub‐threshold PTSD (defined as meeting two or three of the B‐E criteria of the DSM‐5). Only 4% of participants in our study (2 out of 51 for whom we had full access to records) had a diagnosis of co‐morbid PTSD recorded in their clinical notes, and indeed, across the whole Trust, only 39 (1.1%) of 3409 service users with a psychotic disorder diagnosis had a co‐morbid diagnosis of PTSD recorded. See Figure 2.

Based on the PCL‐5, the most prominent PTSD symptoms were negative thoughts and feelings (mean = 12.9, SD = 1.1), followed by hyperarousal (mean = 9.1, SD = 0.8), intrusions (mean = 7.1, SD = 0.7) and avoidance (mean = 3.5, SD = 0.4). The overall mean for the PTSD symptoms across the entire scale was 32.7 (SD = 2.6).

**FIGURE 3:**
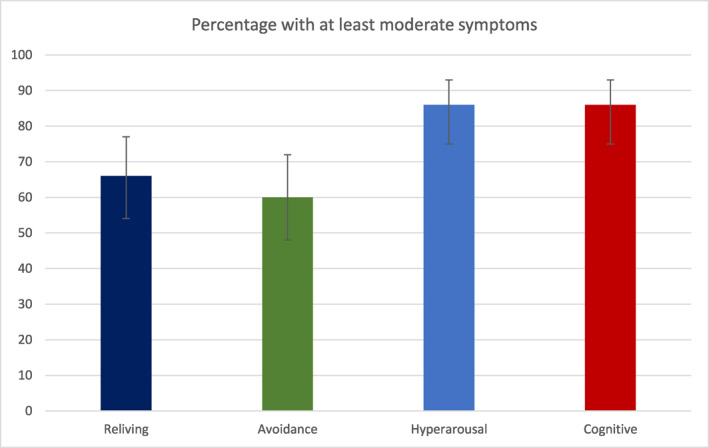
Percentage of participants (and 95% CI) who score 1 or more symptoms as moderate or more severe, by symptom domain.

More than 80% of study participants scored at least moderate on hyperarousal (86%, *n* = 51) and negative thoughts and feelings (86%, *n* = 51), and more than 60% scored at least moderate on intrusive experiences (66%, *n* = 43) and avoidance (60%, *n* = 39). Overall, the percentage of people who scored at least moderate on any symptoms of PTSD was 90% (*n* = 61). See Figure 3.

### Trauma, Mental Health Difficulties and Interest in Receiving Trauma‐Focused Therapy

Sixty‐five percent (*n* = 44, 95% CI 54%, 76%) of study participants believed that their mental health difficulties were related to a past trauma, while 21% (*n* = 14) believed they were not related, 9% (*n* = 6) were unsure, and 6% (*n* = 4) had missing data.

When we asked participants if they would want to receive a trauma‐focused therapy as a way of helping with their current difficulties, 62% of them (*n* = 42, 95% CI 49%, 73%) said yes, while 29% (*n* = 20) said no or were unsure, and 9% (*n* = 6) had missing data.

The severity of PTSD symptoms was associated with increased interest in receiving a trauma‐focused therapy (OR = 1.07, *p* = 0.001, 95% CI 1.03, 1.11). Of all trauma types, people who experienced sexual abuse were most interested in receiving trauma‐focused therapies (81%), but the confidence intervals for all trauma types overlapped substantially (see Table 3).

**TABLE 3:** Number (%) of people with a trauma history who were interested in receiving TF‐therapy, by trauma‐type

	*N*	Percentage (%)	95% CI
Emotional abuse	33	75	0.60, 0.87
Physical abuse	18	75	0.53, 0.90
Sexual abuse	17	81	0.58, 0.95
Emotional neglect	25	74	0.56, 0.87
Physical neglect	23	72	0.53, 0.86

## DISCUSSION

### Summary of Findings

In this cross‐sectional study almost all participants reported experiencing at least one traumatic event that met DSM‐V criterion A for PTSD, and the large majority were exposed to multiple types of trauma. DSM‐V criteria for PTSD were met in 38% of the sample, with an additional 37% meeting criteria for sub‐threshold PTSD.

Our estimate for prevalence of PTSD in those with a psychotic disorder is within the range reported in schizophrenia in one systematic review (24) and close to the median prevalence estimate of 31% in those with psychotic disorder in another (11). This estimate is far higher than the proportion of people diagnosed with PTSD in the mental health NHS Trust from which our participants were recruited, where only 1.1% of people with a diagnosis of psychosis also had a diagnosis of PTSD. The paucity of recorded PTSD diagnoses that we observed in patients' notes with psychosis diagnosed is consistent with other studies (11).

Likely reasons for the low rate of PTSD diagnosis are: i) that clinicians are not recognising or detecting PTSD in people with psychotic disorder, ii) a perception that a diagnosis of PTSD is less necessary than other diagnoses for the purpose of accessing services or treatment, or iii) a lack of confidence in teasing apart PTSD and psychosis symptoms. Whilst hyper‐arousal and negative thoughts/feelings were the commonest PTSD symptom clusters in our sample, reliving experiences such as intrusive thoughts/images were also common, and seem less prone than other cluster to be misattributed to or overshadowed by a psychosis diagnosis.

Where traumatic experiences and PTSD symptoms arise before the onset of a psychotic disorder, it is plausible that the onset and maintenance of psychotic experiences are caused by inadequately processed traumatic memories (25). High levels of emotion during a traumatic experience, and mechanisms such as dissociation to help manage this, could lead to fragmentation of memories that give rise to psychotic phenomena (26). For example, intrusions could be experienced as hallucinations if they are not recognized as trauma memories and attributed externally instead (27). However, more research is required to better understand how traumatic experiences result in psychotic phenomena (28).

The majority of participants in our study believed their mental health problems were linked to their past traumatic experiences, consistent with trauma‐based models of psychosis development (34) rather than biological models of causation that have dominated the field over many decades and that have sometimes led to the dismissal of such views.

Around two thirds of participants in our study were interested in trauma‐specific therapy, with interest in receiving therapy being stronger in those who reported more severe PTSD symptoms. Given that PTSD appears to both increase the risk of developing psychosis and worsen its outcome, and that service users express a desire to engage with trauma‐specific therapy as we demonstrate here, it follows that an evidence base evaluating trauma‐focused psychological interventions (that are NICE‐recommended treatments for PTSD) is needed in people with ARMS or psychotic disorders.

The results of recent systematic reviews suggest that trauma‐focused treatments are effective at improving symptoms of PTSD in people with psychosis (29, 30) and that they have a small effect on reducing the positive symptoms of psychosis, although evidence for the latter is less consistent (31). Further trials of trauma‐focused therapies to prevent the onset of or improve outcomes in psychosis are required (32, 33).

### Strengths and Limitations

The strengths of this study are that we recruited participants from various NHS clinical teams to gain representation of patients with psychosis across the whole spectrum of secondary care service, and our sample size is large enough to be adequately powered to estimate the proportion of people with trauma and PTSD symptoms with reasonable precision. Furthermore, we supplemented this by exploring patient views on the relationship between trauma and psychosis, as well as views on receiving trauma‐focused therapy that have rarely been described in the literature.

However, our study also has a number of limitations. First, as this was a cross‐sectional study it is not possible to rule out the possibility that trauma exposure and subsequent PTSD symptoms occurred secondary to the presence of a psychotic illness. However, the age of trauma exposure and date of diagnosis suggest that this is not the case in most instances, and that PTSD symptoms pre‐dated the onset of psychotic phenomenology. Second, our response rate estimate of 62% means it is possible that our estimates of trauma exposure and prevalence of PTSD might be overestimated if service users without a history of trauma were less likely to respond than those with such a history. It is also possible that other elements of our recruitment process, such as the use of clinicians as well as non‐clinicians with recruitment, may have influenced who participated in our study and the estimates we observed. Third, we used the PCL‐5 self‐report measure of PTSD symptoms rather than the CAPS semi‐structured interview that is the gold standard for assessing PTSD; hence it is possible that we over‐estimate prevalence of PTSD in our study. However, the PCL‐5 is a well‐validated tool for assessing PTSD, whilst a systematic review of undetected PTSD in secondary care mental health services suggested estimates of PTSD were similar for self‐report and interview‐based measures, with type of assessment explaining little of the heterogeneity in estimates (11).

### Implications

Our study highlights the wide range of traumatic events that service users may link to their psychotic experiences, from childhood abuse to bereavement in adult life. A focus on interventions that target these traumatic life events through memory processing therapies, such as EMDR or TF‐CBT, might provide us with new therapeutic avenues for a substantial subgroup of people with psychosis. Consistent with this, the majority of patients in our study believed there was a clear link between their trauma and their psychotic symptoms, and most expressed an interest in receiving a trauma‐focused therapy (27, 34).

It is also clear that the current reality for people with psychosis in secondary care mental health services is that PTSD is often undetected (11). This underlines the need for increased awareness and responsibility on mental health services to identify and address the effect of trauma on service users, as has been previously highlighted (35, 36, 37), and to evaluate approaches for trauma‐informed care (38).

## CONCLUSIONS

Trauma exposure and subsequent PTSD symptoms are very common among people with psychotic disorder or ARMS. Most participants in our sample had experienced multiple traumatic events and believed that trauma was a causal factor for their mental health problems. In addition, most participants were interested in psychological therapy targeting their traumas as a treatment option. Our study highlights the need for evaluation of trauma‐focused interventions to improve outcomes in this patient group.

## Supporting information

Supporting Information S1
