# Supplementary material for: Examining the Relationship Between Trauma, Post‐Traumatic Stress Disorder and Psychosis in Patients in a UK Secondary Care Service
Source: Psychiatr Res Clin Pract. 2023 Jan 20;5(2):51–9. doi: 10.1176/appi.prcp.20220028 (PMC10245461; doi:10.1176/appi.prcp.20220028)
Supplement: Supplementary file 1 — Supporting Information S1 [file RCP2-5-51-s001.docx]

**Post-traumatic stress disorder (PTSD) in people with psychosis and people at increased risk of developing psychosis**

*Supplementary document*

CTQ questions used in this study

1. “I knew that there was someone to take care of me and protect me” (physical neglect)
2. “There was someone in my family who helped me feel that I was important or special” (emotional neglect)
3. “I felt loved” (emotional neglect)
4. “People in family hit me so hard that it left me with bruises or marks” (physical abuse)
5. “People in my family said hurtful or insulting things to me” (emotional abuse)
6. “I got hit or beaten so badly that it was noticed by someone like a teacher, neighbour, or doctor” (physical abuse)
7. “I felt that someone in my family hated me” (emotional abuse)
8. “Someone tried to touch me in sexual way, or tried to make me touch them” (sexual abuse)
9. “There was someone to take me to the doctor if I needed it” (physical neglect)
10. “I believe that I was sexually abused” (sexual abuse)
